# Not the presence but the timing of acoustic signals influence dogs’ behaviour toward an artificial agent

**DOI:** 10.1038/s41598-025-01880-9

**Published:** 2025-05-14

**Authors:** Judit Abdai, Zsófia Lévai, Zsuzsanna Gedai, Ádám Miklósi

**Affiliations:** 1 Department of Ethology, Eötvös Loránd University, Budapest, Hungary; 2 Center for Mind/Brain Sciences, University of Trento, Rovereto, Italy; 3 HUN-REN-ELTE Comparative Ethology Research Group, Budapest, Hungary

**Keywords:** Dog-robot interaction, Communication, Congruency, Acoustic signal, Vocalization, Animal behaviour, Social behaviour

## Abstract

Some features of communicative signals may only direct the attention of the receiver to the signaller, and others may convey specific aspects of the message. Dogs rapidly engage in complex interactions with artificial agents allowing to test whether auditory cues of these agents can only serve as attention-getting cues or they can also convey context-specific message. Dogs encountered the agent first when it helped them to obtain an unreachable reward (Problem-solving); the agent was either (a) silent, or it emitted sound (b) at specific time intervals (Incongruent), or (c) congruently with its interactive behaviours (Congruent). Then, dogs could obtain a reward by following the agent’s indication between two hiding places (Two-way choice). Dogs in all groups displayed communicative behaviour toward the agent. However, the Congruent group started to gaze at the agent later at the beginning of the Problem-solving, and their choice was at chance level in the Two-way choice. No difference was found between the Silent and Incongruent groups. Thus, not the attention-getting aspect of the sound, but the temporal synchronicity of the sound and specific behaviour influenced dogs’ behaviour.

**Supplementary Information:**

The online version contains supplementary material available at 10.1038/s41598-025-01880-9.

## Introduction

Communication rests on complex deployment of mutual signalling between the sender and the receiver. It is the senders’ main interest that their signals effectively influence the receivers’ behaviour. This aim is achieved by evolving and/or developing signals with specific design features, for example, effective signals may be redundant, conspicuous, multimodal, repetitive, etc. (see e.g.,^1^. Some features of the signal may be deployed by the sender to direct the attention to the signaller, while others may signify specific aspects of the message. For example, cluck calls produced by the hen help the chicks to localise their mother, while food calls of the males accompanied by appropriate visual signals direct the females’ attention to the grains on the ground (e.g.,^2^.

Studies involving robots as social partner can contribute to our understanding of communication, by providing a new model to study whether and how adding modalities to a signal increases its effectiveness or whether congruency of signal features facilitates the communicative interaction^3^. The role of specific features of communicative signals also becomes significant if one aims to design artificial agents endowed with effective communication abilities. Many robots are designed to engage in long-term interaction with humans or other agents (e.g., pets) which may require not only the robot to carry out specific actions, but also to provide feedback and communicate with the agent. However, currently the required complexity of effective communication, and how it changes based on specific contexts is unclear.

Relying on dogs’ communicative behaviour in this context could be advantageous because companion dogs are typically used to communicate with members of another species (humans) (e.g.,^4^ and there is also evidence that they rapidly develop effective interaction with artificial agents (unidentified moving object; UMO) (e.g.,^5,6^. In previous dog-robot interaction studies, the main focus was on how different motion cues and/or visual signals of the UMO influence dogs’ behaviour. However, since acoustic features play an important role both in dog-dog and dog-human interactions, it is an open question to what extent this modality enhances interaction in a dog-robot dyad. The possibility of manipulating the signalling behaviour of the UMO also allows us to separate attentional and communicative aspects of the signal. These potential differences can be traced back to the issue of congruency between signal components (see similar concept for human communication, e.g.,^7^. Strictly congruent components (synchronised in time) may increase the communicative function of the signal, while incongruent components (features displayed in an asynchronous way) may only increase the attention-getting effect. In humans, exposure to congruent co-speech gestures support multi-modal language comprehension^8^. Findings show that dogs also rely on congruency in communication. Colbert-White et al.^9^ found that dogs follow human auditory cues to find a hidden reward when they occur in parallel to a pointing gesture, despite the observation that when verbal and visual commands contradict each other, dogs preferentially carry out the action indicated with a gesture^10^. Albuquerque et al.^11^ also showed that dogs expect the emotional valence of vocalization and facial expression to match both in dogs and humans, and Faragó et al.^12^ found that dogs have a mental representation of the size of the caller based on its vocalization. Congruent signal feature can be envisaged also as being redundant, nevertheless, they may assist the receiver to direct its attention to the most relevant part of the signalling behaviour rather than attending the signal per se.

In the present study, we investigated whether the UMO displaying auditory cues congruently with specific events in a problem-solving/helping situation facilitates the interaction. We applied similar procedure as Gergely et al.^6^ consisting of two phases. In the Problem-solving phase, the UMO helped the dogs to retrieve an inaccessible hidden food (six trials), which was followed by a Two-way choice phase where the UMO provided directional information about the location of a hidden food reward (16 trials).

Importantly, dogs assigned to different experimental groups encountered a UMO endowed with altered acoustic signalling behaviours in the problem-solving situation (in the Two-way choice phase, the UMOs’ behaviour was the same in all groups). While the UMO carried out the same reactive and helping behaviour in the case of all subjects, in one (control) group of dogs the UMO was silent (Silent group), whereas in the other two it emitted artificial sounds (Incongruent and Congruent groups). To be able to disentangle whether saliency of the sound or its communicative relevance is important, in the Incongruent group the UMO emitted a sound at specific time intervals (irrespectively of its action and the context) while moving to the location of the unreachable food, whereas in the communicative, Congruent group, it only displayed sound in parallel with specific actions (e.g., starting to move, when the dog approached it, when it got close to the location of the hidden food).

To avoid any confounding effect of previous experience, the UMO was equipped with artificial sounds developed by Korcsok et al.^13,14^. The forming of these sounds relied on simple encoding rules that are general across terrestrial vertebrates: their fundamental frequency and call length. Human participants found sounds with low fundamental frequency and short call length as having positive valence, and those with high fundamental frequency as more intense^13^. Further, in an online questionnaire, humans were more likely to indicate approaching behaviour to an unidentified agent/object if it elicited short calls, and long calls with low pitch or low pitch with no formants, whereas they indicated avoidance in the case of low pitch with formants or long calls with high pitch^14^.

We expected that dogs display more thorough communicative behaviour (gazing at the UMO earlier and displaying more frequent gaze alternations between the UMO and the hiding location) when the UMO emits sounds, compared to when it is silent. In addition, we assumed that the dogs show more frequent communicative interactions toward the UMO which emits sounds that are congruent with its behaviour (as if the UMO communicated with the dogs) compared to the incongruent UMO when there was no correlation between the actions and the sound production.

We expected that dogs find the hidden food more often based on the agent’s behaviour in the two-way choice task when they had encountered the congruent UMO in the Problem-solving phase. This would provide evidence that after short exposure, dogs may be able to generalise the function of congruent communicative vocalisations to a new context. Alternatively, dogs’ performance in the two vocal groups do not differ because in the Congruent group the sounds were emitted in a too broad context (no specific sound was associated with the food or a particular action) and/or any sound emitted during the choice task may overshadow the dogs’ experience in the problem-solving task.

## Results

### Problem-solving phase

We measured the latency of dogs’ first look at the UMO partner before the UMO started to move, and the frequency of alternating their gaze between the location of the unreachable food and the UMO (for the looking duration toward the UMO and time spent near it, see Supplementary Information). We measured in all cases whether dogs’ behaviour differed in the three groups (Silent, Incongruent, Congruent) in the first versus second half of the phase (Trial section 1: trials 1–3; Trial section 2: trials 4–6).

**Fig. 1:**
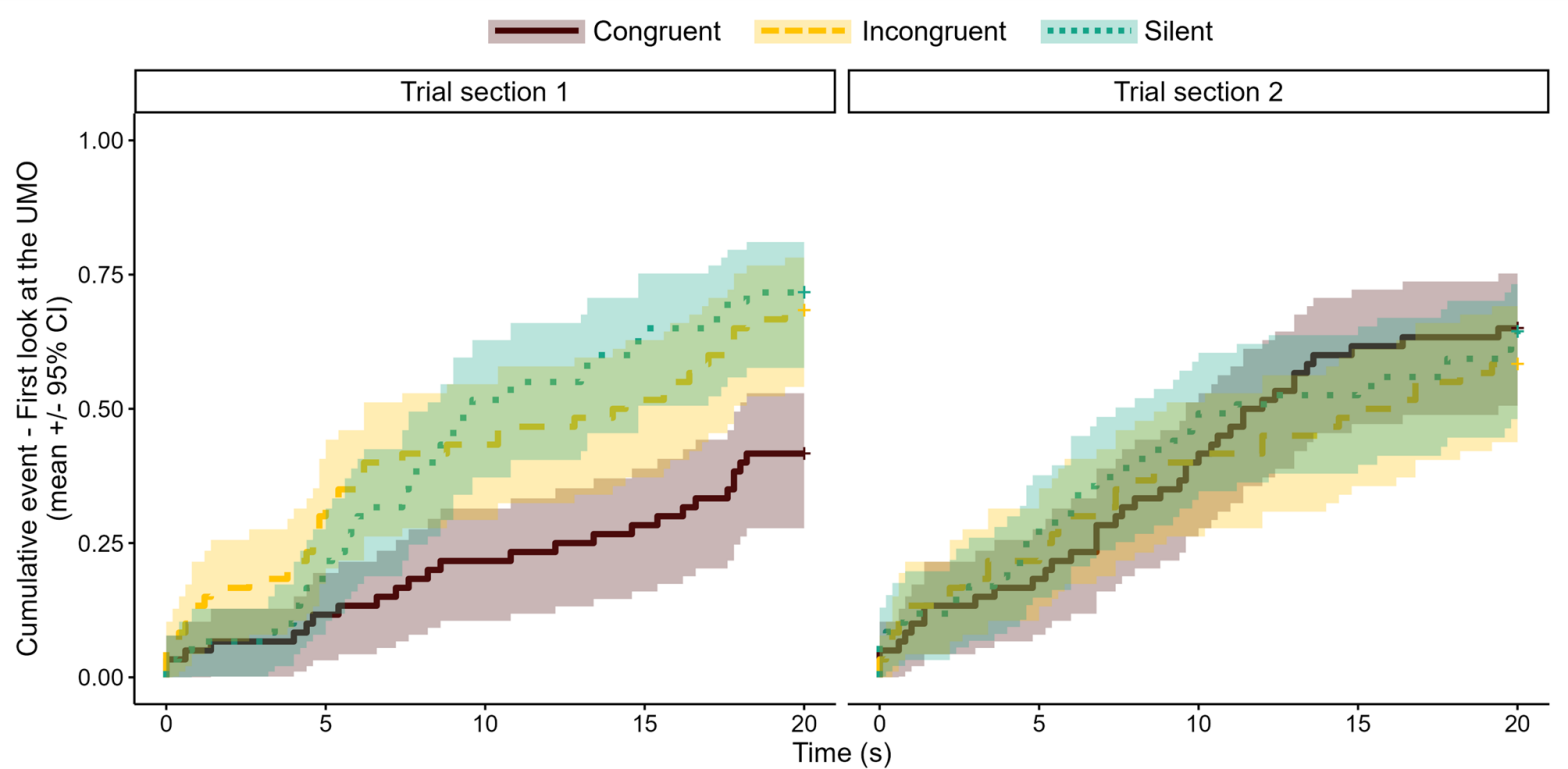
Latency of first look at the UMO in the Congruent, Incongruent and Silent groups in the first and second half of the trials of the Problem-solving phase.

We found that the two-way interaction of group and trial section had a significant effect on the latency to first look at the UMO after the owner released the dog (mixed-effects Cox regression: $\:{\chi\:}_{2}^{2}$ = 10.498, *p* = 0.005) (Fig. 1). Pairwise comparison showed that although in the second half of the trials there was no difference between the three groups (Trial section 2: Congruent vs. Incongruent, β ± SE = 0.134 ± 0.342; *p* = 0.919; Congruent vs. Silent, β ± SE = 0.022 ± 0.339, *p* = 0.998; Incongruent vs. Silent, β ± SE = -0.112 ± 0.343, *p* = 0.943), at the beginning, dogs in the Congruent group gazed at the UMO later than dogs in the Incongruent and Silent groups (Trial section 1: Congruent vs. Incongruent, β ± SE = -0.873 ± 0.357; *p* = 0.039; Congruent vs. Silent, β ± SE = -0.966 ± 0.357, *p* = 0.019; Incongruent vs. Silent, β ± SE = -0.093 ± 0.333, *p* = 0.958). We further found that dogs in the Congruent group looked at the UMO sooner in Trial section 2, than in Trial section 1, whereas no difference was found in the other two groups (Trial section 1 vs. 2: Congruent, β ± SE = -0.857 ± 0.263; *p* = 0.001, Incongruent, β ± SE = 0.150 ± 0.238; *p* = 0.527; Silent, β ± SE = 0.131 ± 0.230; *p* = 0.569).

Regarding the frequency of gaze alternation, we did not find difference between the three groups (GLMM with negative binomial distribution: Group, $\:{\chi\:}_{2}^{2}$ = 0.377, *p* = 0.828) and the two trial sections (Trial section, $\:{\chi\:}_{1}^{2}$ = 3.625, *p* = 0.057; Group x Trial section, $\:{\chi\:}_{2}^{2}$ = 1.328, *p* = 0.515).

### Two-way choice phase

In this phase, a food was hidden in one of two hiding locations and the UMO partner indicated the baited location to the subjects. We assessed dogs’ success of finding the food (1: dog’s head is getting within 10 cm of the baited, 0: dog’s head is getting within 10 cm of the non-baited location), and tested whether there is a difference in dogs’ success in the three groups, and whether their behaviour changed throughout the phase.

**Fig. 2:**
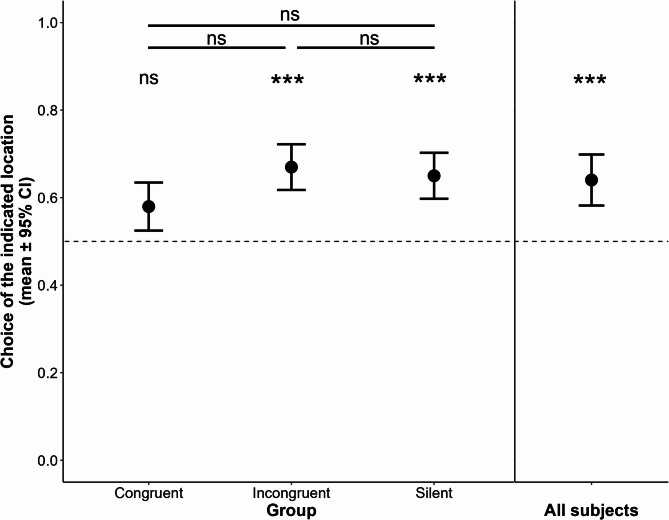
Dogs’ choice performance did not differ between groups or across trials. Overall, dogs performed significantly above chance level; regarding the groups, dogs’ performance was above chance level in the Incongruent and Silent groups, but not in the Congruent group. *** *p* < 0.001.

We found that dogs’ success was not influenced by the previous behaviour of the UMO (GLMM with binomial distribution: Group x Trial section, $\:{\chi\:}_{6}^{2}$ = 11.641, *p* = 0.070, Group, $\:{\chi\:}_{2}^{2}$ = 3.506, *p* = 0.173), and their performance did not change during the trials (Trial section, $\:{\chi\:}_{3}^{2}$ = 5.228, *p* = 0.156). Dogs’ performance overall was significantly above chance level (*N* = 60, z = -13.519, *p* < 0.001); however, when assessed separately for the three groups, we found that dogs’ performance was above chance level in the Incongruent and Silent groups (Incongruent: *N* = 20, z = -3.376, *p* < 0.001; Silent: *N* = 20, z = -3.464, *p* < 0.001), but not in the Congruent group (Congruent: *N* = 20, z = -1.726, *p* = 0.084) (Fig. 2).

## Discussion

Similarly to previous dog-UMO interaction studies (e.g.,^15,16^, we found that dogs successfully interacted with a UMO even without it using acoustic signals; however, the vocalization of the UMO influenced dogs’ behaviour in a novel way. In the Problem-solving phase, we found that dogs in all three groups displayed gaze alternation between the UMO and the cage with similar frequency. Although dogs in the Congruent group started to gaze at the UMO later than dogs in the Silent and Incongruent groups, this difference disappeared by the end of this phase. Also, although dogs successfully followed the indication of the UMO in the two-way choice task and there was no significant difference between groups, detailed analysis revealed that dogs’ performance in the Congruent group was at chance level. Thus, dogs’ behaviours were more alike in the Silent and Incongruent groups throughout the experiment, indicating that the results do not reflect the effect of the saliency of an auditory cue, but dogs consider the timing of the cues as well.

Based on the results one may conclude that dogs in the Congruent group were less likely to regard the UMO as a social partner than dogs in the other two groups, or the difference in the frequency of emitted sound between the Congruent and Incongruent groups could influence the result. Thus, dogs might be distressed by the sound, attending more to this UMO, or looking at the UMO more rapidly over time because they needed time to habituate to the sound. These could have led to decreased (i.e., chance-level) performance during the two-way choice task as well. However, frequency of gaze alternation between the UMO and the cage, looking duration at the UMO and time spent near the UMO (see Supplementary Information) did not differ between groups in either of the sections of the Problem-solving phase. Based on this, it seems that dogs did not show avoidance (or approach) due to the more/less sounds emitted by the UMO (or even due to the presence or absence of it).

We offer an alternative explanation. It is possible that dogs in the Congruent group have learnt that the production of a vocalisation predicts the UMO’s approaching behaviour when encountering a problem. Thus, these dogs could maintain their interest in watching the unreachable reward because there was no need to check the UMO’s behaviour visually^17,18^. Dogs in the Congruent group looking later or not at all at the UMO resulted in the UMO only starting to help after a longer time (up to 20 s), which explains why these dogs eventually started to gaze at it sooner as well. This change in behaviour was not observed in dogs of the Incongruent group thus, it seems that the sound effect alone was not sufficient for dogs to rely on (i.e., hearing the sound did not indicate that help is on the way despite the UMO always emitting a sound 2 s after starting to move). This does not necessarily indicate the understanding of the sounds as communicative signals, rather it is possible that predictability of the sound-action pattern could result in such behaviour in the Congruent group.

Dogs in the Congruent group displayed similar frequency of gaze alternation as the other two groups, that is, a behaviour described as communicative behaviour in problem-solving contexts^17,18^. One may suggest that in our specific case this may indicate more that the dog is being cautious around the UMO, but considering the lack of difference between groups and that there is no decrease/increase in looking duration toward or time spent near the UMO, we suggest that this behaviour is likely to be a communicative signal in our case as well. Considering that there were no differences between groups, we suggest that the UMO’s helping behaviour alone led to this communicative behaviour on the part of the dog.

In the Two-way choice phase, the sound was displayed right before the indication of the correct bowl in all groups. This could direct the visual attention of dogs to the UMO only due to its saliency, but if dogs consider it as communication, it can be perceived as an ostensive cue. Although ostensive cues displayed by a human partner before a pointing task can facilitate dogs to perform well in two-way choice tasks (see e.g.,^19,20^, it has also been described that in specific cases, ostensive cues may hinder dogs’ performance in choice tasks due to directing the focus away from the task (e.g.,^21,22^. Colbert-White et al.^9^ also suggested that dogs in their study might have interpreted cues easier due to the lack of effort to call dogs’ attention, thus minimizing the distracting information. In our case, the UMO being a novel partner (compared to regular partners such as a human or a dog) and thus having to process its social behaviour (including not only motion cues but acoustic ones as well) might have directed dogs’ attention more on the partner rather than the choice task, resulting in lower performance in the Congruent group. Although in the Silent and Incongruent groups the partner is novel as well, and in the latter, it even emits sound, here the acoustic cues are random (versus the specific sound-action pattern in the Congruent group) and thus not necessarily add to the complexity of the UMO’s agency.

Associative learning might have played a role in dogs’ behaviour, that is, having a connection between the food and the UMO/sound could facilitate their choice in the Two-way choice phase. However, previous studies showed that dogs interact with UMOs even without it providing them with food reward^5,16^, and in this case, we would have expected dogs in the Congruent group to perform above chance level as well. Another possible explanation can be an attention bias, for example, dogs might pay less attention to the Congruent UMO at the beginning, because the synchronized behaviour and sound was predictable (and thus less surprising). Such congruency could also introduce a bias influencing how and when they gaze at the UMO (and other behaviours) without attributing communicative meaning to the sound display.

As mentioned above, the difference between the Congruent and Incongruent groups could emerge because the former UMOs produced more sounds. To counterbalance the number of sounds between the Congruent and Incongruent groups, we would have needed to increase it in the latter, because in the Congruent group emitting sound was tied to the interaction between the dog and the UMO (that is, modifying the number of sounds was not possible here; see Methods). Due to this, the only option would have been to change the UMO’s behaviour in the Incongruent group (e.g., moving more slowly) to be able to present more sounds in this group. This would have introduced a marked difference in the UMO’s helping behaviour (efficiency) and motion characteristics. Not only this would have influenced the perception of this novel agent as social partner but, for example, delayed retrieval of the food reward could cause distress in the dog (irrespectively of the sound), making it impossible to disentangle if potential change in dogs’ behaviour in this group would be caused by the frequency of sound, inefficiency of the UMO or distress due to delayed reward. Thus, we decided the UMO to have similar behaviour in all groups, resulting in potential differences in the number of sounds (emitting a minimum of two sounds in both groups). However, we suggest that in future studies (using different social context) frequency of sounds should be also controlled for in order to understand its influence on dogs’ behaviour. Note however, that sound alone could not introduce such difference based on our results regarding the Silent and Incongruent group, rather only the frequency of emitted sound. We suggest that if frequency of sound led to distress, habituation, or increased attention to the Congruent UMO, we would have found differences in other behaviours of dogs as well, for example, distress could lead to less time spent near the UMO, and habituation to less time spent looking at the UMO. However, we only found difference between the Congruent and the other groups in the latency of first look at the UMO in the Problem-solving phase, and that dogs in the Congruent group performed on chance level, but not in any other behaviour.

In the present study, we applied artificial sounds created based on biological considerations by Korcsok et al.^13,14^, and we used those that were evaluated as positive or neutral by adult humans^13,14^. Here we focused on the influence of sound display alone vs. congruency of sound display and social behaviour, thus we did not test whether the specific sound has a role in the social interaction. Future studies should explore the perception of these sounds in dogs, that is, whether sound design based on biological rules is important (social vs. non-social sound), and whether the perception of emotion and valence of the sound is similar in dogs as in humans. This information can further contribute to understanding the role of sound display by UMOs in social interactions with dogs.

In the present study, we showed that not the attention-getting aspect of the sound, but rather the temporal synchronicity of the sound and specific behaviour may influence dogs’ behaviour. From this study alone it would be difficult to disentangle if dogs’ reaction was based on the predictable sound-action pattern or dogs attributed further communicative information to the sound displays of the UMO, but the results provide a promising basis for future studies about how vocal cues may contribute to the acceptance of artificial agents as social partners. Further studies are needed to investigate the specific role of vocalisation when encountering an artificial agent, and how the specific sounds used may influence the interaction. Such studies should explore whether dogs also take into consideration the information of the acoustic cues/signals emitted by the UMO, for example, acoustic signals evaluated as positive or negative could provide information to dogs about the place of a hidden food.

## Methods

### Subjects and groups

All methods were approved by the National Animal Experimentation Ethics Committee of Hungary (PE/EA/00032 − 4/2023). All methods were carried out in accordance with relevant guidelines and regulations, the experiment was performed in accordance with the EU Directive 2010/63/EU and all methods are reported in accordance with the ARRIVE guidelines. Owners provided written consent indicating voluntarily allowing their dogs to participate in the study and that the test videos can be used for publication of identifying images in an online open-access publication.

Dogs were semi-randomly assigned to the three groups (counterbalancing age, sex, and breed across groups), that is, dogs were randomly assigned to the three groups in general, but if later a same breed or similar age dog was recruited, they were placed in another group to avoid having only young/old dogs in a group, or several individuals of the same breed in one group. We aimed to have 20 dogs in all groups for the final analysis. We tested 70 dogs above one year of age that could be motivated by food and had no issues with their hearing. We excluded four dogs because they lost their motivation in the Two-way choice phase (i.e., did not make a choice in three consecutive trials or in four trials in total). We excluded one dog because the dog was distressed in the room, and one dog because the dog was not motivated by food. Two dogs were excluded because the owner influenced the dogs’ behaviour in the Two-way choice phase (indicating the baited bowl or stopping the dog before making a choice when it started to approach the non-baited bowl). One dog was excluded because the dog could solve the problem in the Problem-solving phase, and one dog because the UMO indicated the non-baited bowl in the first trial of the Two-way choice phase.

The 60 dogs remaining were assigned to three different groups based on the UMO’s behaviour in the Problem-solving phase. In the *Congruent group*, the UMO emitted sounds at a start of actions and potential interactions (*N* = 20; 11 females; mean ± SD age: 4.8 ± 2.8 years). In the *Incongruent group*, the UMO used the same sounds as in the Congruent group; however, it emitted them in specific time intervals (*N* = 20; 11 females; mean ± SD age: 4.7 ± 2.4 years). In the *Silent group*, the UMO did not display any sounds in the Problem-solving phase (*N* = 20; 11 females; mean ± SD age: 5.2 ± 3.6 years) (for more details about the subjects, see Supplementary Data 1).

### UMO partner and artificial sounds

The UMO was a remote-controlled car (#120090 E10 Michele Abbate GrrRacing Touring Car (LxWxH: 43 cm x 19 cm x 12 cm) controlled by Experimenter (E) 1 from the adjacent room via live camera picture (two Zoom Q2n wide-angle lens cameras). Magnets were attached to the front of the UMO to be able to move the plate in the Problem-solving phase (see below).

Sounds were played by a TWS mini loudspeaker attached on the UMO (under the cover) which connected to an Android 8.0.0. smartphone via Bluetooth (sound was controlled by E2). Due to the small space available for the loudspeaker in the UMO, we had limitations regarding the specific loudspeaker to use. For all subjects, the same loudspeaker set at the same volume was used. As artificial sounds, we used sounds designed by Korcsok et al.^13,14^ that were evaluated as positive or neutral by adult humans^13^, and toward which humans indicated approach behaviour in a questionnaire^14^. We applied five random variations of thirteen sounds with different parameters (overall 65 sounds) with 2 s length. The sounds were displayed in random order during the experiments.

### Procedure

Tests were carried out at the Department of Ethology, Eötvös Loránd University, Budapest, Hungary in two adjacent rooms. In the Problem-solving phase (room size: 6.27 m x 5.4 m), the UMO helped dogs to obtain an unreachable food; depending on the group, the UMO either displayed sounds in communicatively relevant situations, at specific intervals, or not at all. In the Two-way choice phase (room size: 3 m x 5.4 m), dogs had to choose between two hiding places in repeated trials, only one containing food; the UMO indicated the baited place for the dog (the UMO’s behaviour in the three groups were identical in this phase). For a video demonstration of the procedure, see Supplementary Video 1.

#### Problem-solving phase

**Fig. 3:**
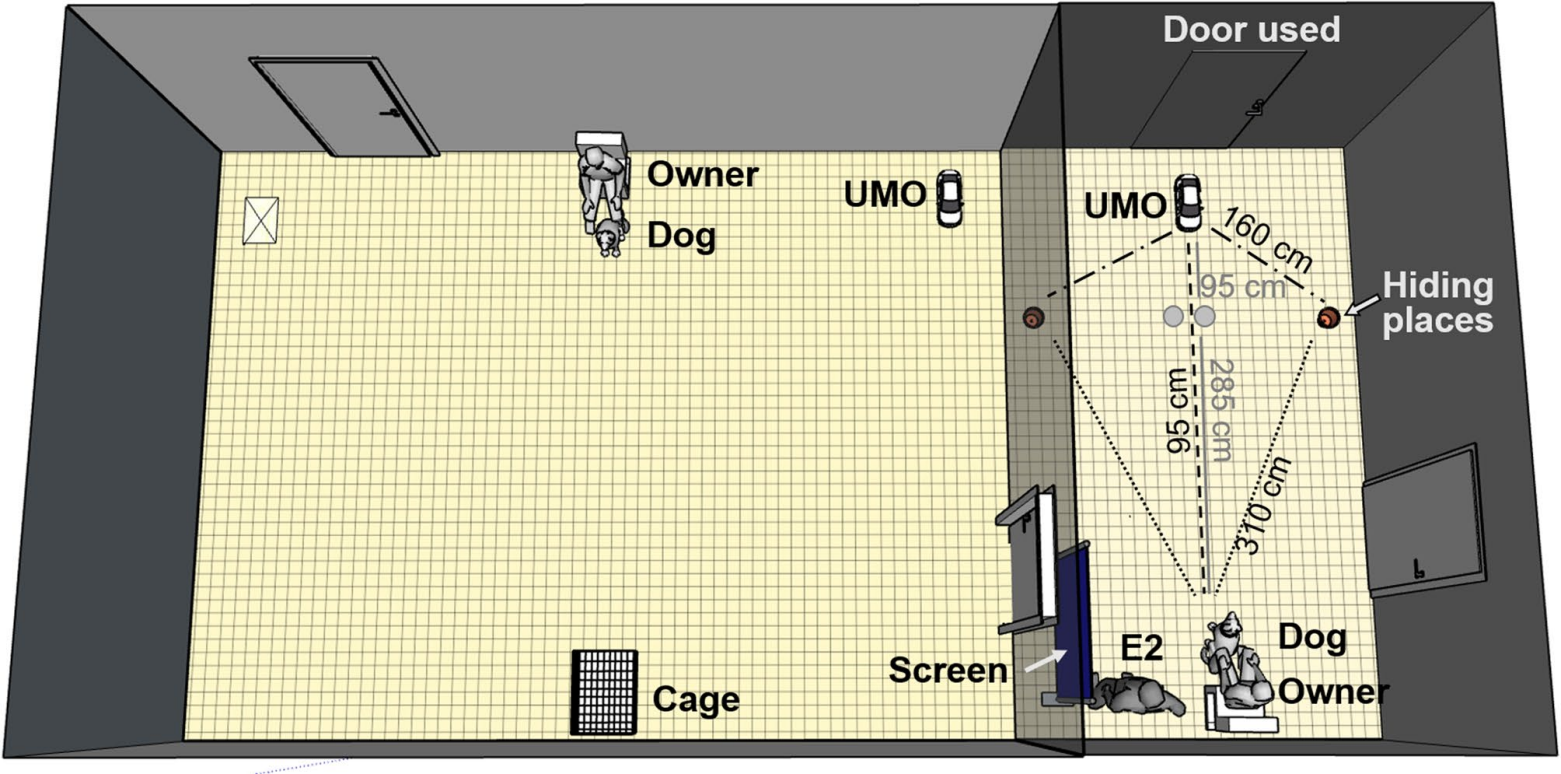
The Problem-solving phase was carried out in the larger test room, whereas the Two-way choice phase in the smaller test room. The ‘X’ in the larger room indicates the other starting point of the UMO. The grey dots in the smaller test room indicate the positions of the hiding places during the warm-up trials.

Before the experiment started, the experimenters placed a cage (LxWxH: 61 cm x 47 cm x 55 cm) and a chair to opposite walls, and the UMO in one of the corners closer to the chair (Fig. 3). The front opening of the cage (WxH: 20 cm x 14 cm) was small enough for dogs not to be able to enter and retrieve the food placed inside. The owner and the dog entered the room along E1; the dog explored the room while E1 gave the instructions to the owner.

First, we familiarized dogs with the UMO. The owner sat on the chair holding the dog in front of him/her, and E1 left the room. Then, the UMO moved around the room once and then stopped at its original position. E1 returned the room, and the owner released the dog. If the dog did not approach the UMO immediately, E1 (and the owner, if needed) approached the UMO and encouraged the dog to approach the UMO as well to ensure that the dog is not afraid to get near the UMO.

Following this, E1 brought in the room two pieces of food (sausage) and a small bowl with metal sheets on its sides (the UMO could attach to these with the magnets on its front to retrieve the bowl). E1 showed a piece of food to the dog while saying the dog’s name, calling its attention; she placed the food in the bowl and put it on the ground. The owner released the dog that ate the food. Then, the owner called the dog back and hold it again. E1 called the dogs’ attention again with a piece of food in her hand, put it in the bowl and placed the bowl inside the cage. E1 left the room, and the owner released the dog. The owner could encourage the dog to try to obtain the food but could not indicate in the direction of the UMO. The UMO started to move either 20 s after the dog was released, or earlier if the dog looked at the UMO. The UMO went to the cage and retrieved the bowl with the food. In the three groups, the UMO behaved differently according to the following description.

In the *Congruent group*, the UMO emitted a sound in situations when a human would have displayed some acoustic signals, that is, the agent vocalised when it performed an action or there was a chance for interaction with the dog. Accordingly, the UMO produced a sound when (a) it started to move, (b) it was approached by the dog within 0.5 m, (c) the dog blocked the entrance of the cage and thus the UMO could not go inside, (d) the UMO got inside the cage, (e) the dog did not approach the UMO after it retrieved the food.

In the *Incongruent group*, the UMO emitted the same sounds as in the Congruent group; however, it emitted a sound 2 s after the UMO started to move and in every 2 s following the end of the previous sound, until the UMO got within 0.5 m of the cage. After the UMO retrieved the food, the UMO emitted a sound only once, if the dog did not approach it within 3 s.

In the *Silent group*, the UMO did not display any sounds.

The overall frequency of vocalisation was (mean ± SD) 3.2 ± 1.4 and 2.4 ± 0.7 in the Congruent and Incongruent group, respectively (Kruskal-Wallis test: $\:{\chi\:}_{1}^{2}$ = 8.983, *p* = 0.003).

After the dog ate the food, the UMO moved to the corner opposite to its starting position. We repeated the procedure overall six times (in the case of one dog only 5 trials were carried out), the UMO changed its location between the two corners in each trial (starting side in the first trial was counterbalanced between dogs). After the last trial, the owner and the dog left the room for a 2–3 min break.

#### Two-way choice phase

Before the owner and the dog entered the small room, the experimenters placed the experimental equipment in the room (Fig. 3). A chair and the UMO were placed to opposite sides of the room, and two empty flowerpots 2.85 m away from the chair, right next to each other in the middle of the room. A screen (WxH: 92 cm x 125 cm) was placed on the left side of the chair.

E2 instructed the owner outside the room. After entering the room, the dog explored the room. Then, the owner put the dog on a 5-metre-long leash, sat on the chair and hold the dog right in front of her. E2 stood next to the chair on its left side. E1 entered the room through the door next to the UMO (only this door was used during the phase) with three pieces of food. In the warm-up trials, we familiarized dogs with moving while being on the leash, and that only one of the pots contain food. E1 showed a piece of food to the dog while saying its name and put it inside one of the pots. The owner released the dog that could eat the food. The owner called the dog back, hold it again, and the same procedure was repeated one more time, E1 placing the food in the other pot (we counterbalanced the sides of the food between dogs).

After the dog ate the second piece of food and the owner hold the dog again, E1 showed a piece of food while saying the dog’s name. Then, E2 placed the screen in front of the dog to block its view of the room. E1 placed the food inside one of the pots and put both pots to the sides of the room near the walls (see Fig. 3), then left the room. E2 took the screen away and stood still tilting her head down. The UMO emitted an acoustic signal (in all groups). About 4 s after the sound stopped, the UMO moved to the baited pot, pushed it a bit, and then returned to its starting position. After the UMO stopped, the owner released the dog. The owner could encourage the dog to look for the food but was not allowed to indicate in any directions to the dog. If the dog did not start to move, the owner could stand up, and move one or two steps forward in a straight line. If the dog chose the baited pot, the dog could eat the food. If the dog chose the non-baited pot, the dog was not allowed to eat the food from the baited pot; the owner called the dog back immediately and could also stop the dog reaching the baited pot by holding the leash.

After the choice, the owner called the dog back, hold it in front of them and E2 put the screen in front of the dog. E1 entered the room with a piece of food and hid it in one of the bowls (and removed the food if the dog chose the non-baited before). The procedure was repeated overall 16 times. The hiding was in a semi-random order, the food could be in the same pot in maximum two consecutive trials (LRRLRLLRRLRLLRRL or RLLRLRRLLRLRRLLR); the starting side was counterbalanced between dogs.

### Behaviour and data analyses

Behaviour of dogs was coded using Solomon Coder version 19.08.02 (https://solomon.andraspeter.com/), and statistical analyses were carried out using R version 4.3.2^23^ in RStudio version 2023.12.1 Build 402^24^. Inter-coder reliability was assessed on a random subsample of dogs (20% of subjects).

In the *Problem-solving phase*, we coded the latency of first look (s) at the UMO after the owner released the dog. Along with the time, we indicated with 1 if the dog looked at the UMO within 20 s, and with 0 if not (using the 20 s maximum time as latency). Spearman correlation indicated acceptable inter-coder reliability (*r*_*s*_ = 0.763, *p* < 0.001). Data was analysed using mixed-effects Cox regression (‘coxme’ package^25^. We also coded the frequency of gaze alternation between the cage and the UMO, after the UMO started to move until it got within 0.5 m of the cage. Inter-coder reliability was acceptable for the frequency of gaze alternation (*r*_*s*_ = 0.731, *p* < 0.001). Based on the AIC values (model comparison with ANOVA; the model with the lowest AIC value was kept, the model was evaluated as better if ΔAIC > 2), negative binomial distribution fit best the frequency of gaze alternation (AIC = 1041.1). Thus, gaze alternation was analysed using generalized linear mixed model (GLMM) with negative binomial distribution (‘lme4’ package^26^. In the case of both models, we carried out backward model selection based on LRT. In the case of the mixed-effects Cox regression, models were compared using ANOVA; in the case of GLMM, we used the drop1 function (‘stats’ package). For non-significant variables, we report the result of the LRT before exclusion from the model; for significant variables, we carried out pairwise comparison with Tukey correction (‘emmeans’ package^27^. In both models, we included the two-way interaction of group (Congruent vs. Incongruent vs. Silent) and trial section (trial 1–3 vs. trials 4–6). Dogs’ ID was included as random factor to control for repeated measurements.

We also analysed the time spent looking at and being near the UMO (see Supplementary Information). To establish if the dogs’ baseline behaviour was similar in the three groups, we analysed separately all these behaviours shown in the first trial of the Problem-solving phase (see Supplementary Information).

In the *Two-way choice phase*, we coded the choice of dogs (dog’s head is getting within 10 cm of the pot), indicating with 1 if the dog chose the baited pot, and with 0 if the dog chose the non-baited one (100% match between coders). Data was analysed using GLMM with binomial distribution; we carried out backward model selection using the drop1 function, based on LRT. We tested the effect of the two-way interaction of group (Congruent vs. Incongruent vs. Silent) and trial section (trials 1–4 vs. trials 5–8 vs. trials 9–12 vs. trials 13–16) on choice. We also tested whether dogs’ performance in the three groups differed from chance level (0.5), using Wilcoxon signed rank test with continuity correction (‘stats’ package). From the analysis of this phase, we had to exclude the data of the last 3 trials of one dog and the last 5 trials of another dog because the UMO indicated the non-baited pot in the previous trial; in the case of two dogs only 15 trials were carried out (see Supplementary Data 1).

We also analysed the time spent looking at the UMO from the moment it emitted a sound until the UMO stopped after the indication (see Supplementary Information).

## Electronic supplementary material

Below is the link to the electronic supplementary material.

Supplementary Material 1

Supplementary Material 2

Supplementary Material 3

## Data Availability

All data are available as Supplementary material.
