# Supplementary material for: Not the presence but the timing of acoustic signals influence dogs’ behaviour toward an artificial agent
Source: Sci Rep. 2025 May 14;15:16692. doi: 10.1038/s41598-025-01880-9 (PMC12078579; doi:10.1038/s41598-025-01880-9)
Supplement: Supplementary file 1 — Supplementary Material 1 [file 41598_2025_1880_MOESM1_ESM.pdf]

Supplementary information for:

Not the presence but the timing of acoustic signals influence dogs' behaviour toward an artificial agent

By J Abdai, Z Lévai, Z Gedai, Á Miklósi

in *Scientific Reports*

Latency of first look at the UMO and frequency of gaze alternation in the first trial of the Problem-solving phase

We analysed the latency of first look at the UMO after the owner released the dog, separately in the first trial of the Problem-solving phase, to assess if there was a basic difference between the dogs tested in the three groups. We indicated with 1 if the dog looked at the UMO within 20 s, and with 0 if not (using the 20 s maximum time as latency). Data was analysed using Cox regression ('survival' package[1,2]). We found that the group did not have a significant effect on the latency to first look at the UMO in the first trial of the Problem-solving phase (Cox regression: Group, χ^2_2 = 3.095, p = 0.213).

We also compared between groups the frequency of gaze alternations between the UMO and the cage, in the first trial of the Problem-solving phase. We used generalized linear model with negative binomial distribution (GLM) (based on the AIC value the negative binomial and poisson distribution both fit the data; based on the suggestion of the 'check_distribution' function ('easystats' package[3]). Group did not influence the frequency of gaze alternation between the UMO and the cage in the first trial of the Problem-solving phase (GLM with negative binomial distribution: Group, χ^2_2 = 0.265, p = 0.876).

Looking duration at the UMO in the Problem-solving phase

We analysed the percentage of dogs' looking duration at the UMO from the moment the UMO started to move until it got within 0.5 m of the cage. For the intercoder reliability analysis, we exported the specific sections of the coding sheets of both coders from the Solomon Coder, and checked the correspondence between coders for all frames (5 frames per second). Inter-coder reliability was tested calculating Cohen's kappas and we provide the percentage of agreement

as well (mean κ ± SD = 0.624 ± 0.369, mean % ± SD = 79.96 ± 25.10). We analysed the data using linear mixed model (LMM; 'lme4'package[4]). The residuals of the model were normally distributed (Kolmogorov-Smirnov test: D = 0.047, p = 0.404). We used the drop1 function ('stats' package) for backward model selection. For the selection, we used likelihood ratio test (LRT), the non-significant variables were excluded from our model step-by-step (results of LRT are reported before exclusion). For the significant variables in the final model, we carried out pairwise comparison (Tukey adjustment; 'emmeans' package[5]) and we report the contrast estimates (β ± SE). We included the two-way interaction of group (Communicative vs Non-communicative vs Silent) and trial section (trial 1-3 vs trials 4-6). Dogs' ID was included as random factor to control for repeated measurements. We also analysed the data within the first trial to assess if dogs' behaviour had a basic different in the three groups, using linear model (Kolmogorov-Smirnov test: D = 0.105, p = 0.487).

Dogs' looking duration at the UMO did not differ between groups (LMM: Group x Trial section, χ^2_2 = 2.357, p = 0.307; Group, χ^2_2 = 1.142, p = 0.565), but overall dogs looked at the UMO longer in Trial section 2 than in Trial section 1 (Trial section, χ^2_1 = 9.730, p = 0.002; Trial section 1 vs 2, β ± SE = -5.870 ± 1.870, p = 0.002). Regarding the first trial, we did not find difference between the three groups (LM: Group, χ^2_2 = 1.393, p = 0.498).

Time spent near the UMO in the Problem-solving phase

We analysed the percentage of time dogs spent within 0.5 m of the UMO, from the moment the UMO started to move until it got within 0.5 m of the cage. Inter-coder reliability analysis was carried out the same way as described above (mean κ ± SD = 0.559 ± 0.384, mean % ± SD = 78.5 ± 26.5). Data distribution was determined by using the check_distribution function ('easystats' package[3]), suggesting tweedie distribution. Generalized linear mixed models (GLMMs) were fitted using the glmmTMB function ('glmmTMB' package[6]). Model selection was carried out in the same way as described above. We also analysed the data within the first trial to assess if dogs' behaviour had a basic different in the three groups, using linear model (Kolmogorov-Smirnov test: D = 0.151, p = 0.129).

The time spent near the UMO did not differ between groups (GLMM with tweedie distribution: Group x Trial section, χ^2_2 = 0.553, p = 0.758; Group, χ^2_2 = 0.279, p = 0.870), but overall dogs spent more time near the UMO in Trial section 2 than in Trial section 1 (Trial

section, χ^2_1 = 6.978, p = 0.008; Trial section 1 vs 2, β ± SE = -0.280 ± 0.105, p = 0.008). Regarding the first trial, we did not find difference between the three groups (LM: Group, χ^2_2 = 0.081, p = 0.961).

Looking duration at the UMO in the Two-way choice phase

We analysed the percentage of dogs' looking duration at the UMO from the moment the UMO emitted the sound until it stopped at its starting location after the indication of the baited pot. For the intercoder reliability analysis, we exported the specific sections of the coding sheets of both coders from the Solomon Coder, and checked the correspondence between coders for all frames (5 frames per second). Inter-coder reliability was tested calculating Cohen's kappas and we provide the percentage of agreement as well (mean κ ± SD = 0.703 ± 0.153, mean % ± SD = 86.13 ± 7.78). We analysed the data using linear mixed model (LMM; 'lme4'package[4]). The residuals of the model were normally distributed (Kolmogorov-Smirnov test: D = 0.030, p = 0.371). We used the drop1 function ('stats' package) for backward model selection. For the selection, we used likelihood ratio test (LRT), the non-significant variables were excluded from our model step-by-step (results of LRT are reported before exclusion). For the significant variables in the final model, we carried out pairwise comparison (Tukey adjustment; 'emmeans' package[5]) and we report the contrast estimates (β ± SE). We included the two-way interaction of group (Communicative vs Non-communicative vs Silent) and trial section (trial 1-3 vs trials 4-6), the two-way interaction of group and pot (baited vs non-baited), and the two-way interaction of trial section and pot. Dogs' ID was included as random factor to control for repeated measurements.

Dogs' looking duration at the UMO in the Two-way choice phase did not differ between groups or trial sections (LMM: Group x Trial section, χ^2_6 = 8.263, p = 0.220; Group x Pot, χ^2_2 = 2.587, p = 0.274; Trial section x Pot, χ^2_3 = 3.552, p = 0.314; Group, χ^2_2 = 2.537, p = 0.281; Trial section, χ^2_3 = 2.148, p = 0.542). However, we found that dogs that chose the baited pot looked longer at the UMO than dogs that chose the unbaited one (Pot, χ^2_1 = 5.515, p = 0.019).

References

1. Therneau, T. A package for survival analysis in R. Preprint at https://CRAN.R-project.org/package=survival (2023).
2. Therneau, T. M. & Grambsch, P. M. *Modeling Survival Data: Extending the Cox Model*. (Springer, New York, 2000).

3. Lüdecke, D. *et al.* easystats: Framework for easy statistical modeling, visualization, and reporting. Preprint at https://easystats.github.io/easystats/ (2022).

4. Bates, D., Maechler, M., Bolker, B. & Walker, S. Fitting linear mixed-effects models using lme4. *J Stat Softw* **67**, 1–48 (2015).

5. Lenth, R. V. emmeans: Estimated Marginal Means, aka Least-Squares Means. Preprint at (2024).

6. Brooks, Mollie, E. *et al.* glmmTMB balances speed and flexibility among packages for zero-inflated generalized linear mixed modeling. *R J* **9**, 378 (2017).
